# Self-Care Behaviors and Associated Factors Among Hypertensive Patients at Dessie Referral Hospital, Northeast Ethiopia

**DOI:** 10.1155/ijhy/1774636

**Published:** 2025-04-24

**Authors:** Assen Muhe, Mesfin Haile Kahissay, Mohammed K. Ali, Solveig A. Cunningham, Bruck Messele Habte

**Affiliations:** ^1^Department of Pharmacy, College of Medicine and Health Sciences, Wollo University, Dessie, Ethiopia; ^2^Department of Pharmaceutics and Social Pharmacy, School of Pharmacy, College of Health Sciences, Addis Ababa University, Addis Ababa, Ethiopia; ^3^Department of Global Health, Rollin School of Public Health, Emory University, Atlanta, Georgia, USA

**Keywords:** Dessie, Ethiopia, hypertension, self-care behavior, self-care practice

## Abstract

**Introduction:** Hypertension poses a significant global health challenge, leading to serious health conditions and premature death. Effective blood pressure control is often hindered by patients' nonadherence to self-care behaviors. This study evaluates these behaviors and their influencing factors among hypertensive patients at Dessie Referral Hospital, Ethiopia.

**Methods:** Conducted from October 20 to November 30, 2019, this mixed-methods study involved 370 hypertensive patients from the hospital's outpatient clinic. Data were collected via structured questionnaires and analyzed using multivariable logistic regression. Additionally, 14 in-depth interviews provided qualitative insights, analyzed thematically.

**Results:** Only 29.4% of patients fully adhered to self-care recommendations. Urban dwellers showed 70% less adherence than rural counterparts. Adherence varied with the duration since diagnosis, with medium-duration patients being less likely to adhere. Interviews revealed personal strategies for managing diet, exercise, medication, and lifestyle, highlighting the struggle with adherence and innovative solutions to challenges.

**Conclusion:** Adherence to self-care among hypertensive patients is alarmingly low, influenced by diagnosis duration, residency, and BMI. Addressing hindrances like living conditions, work, cultural norms, and peer influence is vital. Healthcare providers must focus on education that promotes behavior change and support. Patient engagement in self-care is essential. Future research should investigate healthcare organizational and provider influences. Implementing these strategies could markedly improve hypertension management and patient outcomes.

## 1. Introduction

Hypertension, characterized by a persistent elevation of systolic or diastolic blood pressure equal to or exceeding 140/90 mmHg in adults aged 18 years and older, has evolved into a global health concern. It stands as a significant risk factor for chronic kidney disease, heart failure, myocardial infarction, and premature death, contributing substantially to the burgeoning healthcare costs [1]. Hypertension's ominous impact extends to being a leading cause of stroke, cardiovascular diseases, and global fatalities, with 9.4 million annual deaths attributed to it [2]. In 2013, the World Health Organization (WHO) declared hypertension a “silent killer” and a global public health crisis [3].

The prevalence of hypertension has surged dramatically, affecting approximately one-third of the world's adults, and is projected to reach 1.56 billion by 2025 [1]. In Ethiopia, noncommunicable diseases, including hypertension, account for a significant portion of deaths and disability-adjusted life years (DALYs) lost. Studies within Ethiopia have reported varying rates of hypertension prevalence, such as 18.8% in Sidama Zone [4], 28.3% in Gondar [5], 13.2% in Jimma [6], and 11% in Mekele [7]. Effective self-care behaviors are crucial for controlling blood pressure and mitigating hypertension-related complications. These behaviors encompass medication compliance, regular physical activity, healthy dietary choices, weight management, reduced alcohol consumption, and tobacco avoidance. However, many hypertensive patients struggle to adopt and maintain these essential lifestyle changes [2].

Adopting a combination of dietary practices, reducing sodium intake, engaging in regular aerobic physical activity, limiting alcohol consumption, and maintaining a healthy body mass index (BMI) can significantly reduce blood pressure [1]. Hypertensive patients who adhere to self-care practices experience better blood pressure control, but the lifelong commitment, motivation, and support needed for chronic disease management pose ongoing challenges.

The escalating global prevalence of hypertension underscores the urgency of promoting effective self-care behaviors to combat this public health crisis. This study seeks to address the knowledge gap surrounding self-care practices and their influencing factors among hypertensive patients in Ethiopia, bridging previous research limitations by combining quantitative and qualitative methods to comprehensively explore this vital aspect of hypertension management. The findings will serve as a valuable resource for healthcare professionals, government agencies, and stakeholders, enabling the development of targeted strategies to enhance self-care practices and mitigate the impact of hypertension [1, 2]. This study aims to assess self-care behaviors and related factors among hypertensive patients at Dessie Referral Hospital in Northeast Ethiopia.

## 2. Methods

This study builds upon foundational methods previously discussed in a preprint titled *“Perception about Child Vaccination and its Determinants among Caregivers in Tehuledere Woreda, Northeastern Ethiopia”* [8]. While this preprint influenced the study design and data collection tools, the current research is distinct in its focus on self-care behaviors and associated factors among hypertensive patients at Dessie Referral Hospital.

### 2.1. Study Setting and Period

Dessie, a historic city in Ethiopia and the capital of the South Wollo Zone in the Amhara National Regional State, is located approximately 400 km from Addis Ababa and 475 km from Bahir-Dar, the regional capital. With an estimated population of 245,129 and covering 158.29 km^2^, Dessie is a significant urban center in the region.

Dessie Referral Hospital, established in 1942, is a major healthcare facility offering a wide range of medical services, including care for diabetic and hypertensive patients, surgical procedures, gynecology, dermatology, ophthalmology, pediatrics, and more. The hospital plays a pivotal role, serving a catchment population of approximately 8 million people from multiple regions. It also functions as a training center for healthcare professionals and a teaching site for students from the College of Medicine and Health Sciences at Wollo University. Due to its expertise in chronic care management and the substantial number of hypertensive cases referred from eastern Amhara hospitals, Dessie Referral Hospital was chosen as the study site. The hospital staff comprises 549 members, including 398 health professionals and 151 administrative staff.

Dessie Referral Hospital operates 17 adult outpatient departments, including two dedicated to chronic care. One unit serves both hypertensive and diabetic patients, while the other caters to hypertensive and heart failure patients. The hospital conducts daily patient education programs on hypertension and diabetes management before physician consultations. On average, each outpatient department attends to 25 hypertensive patients daily, with appointment intervals varying from 1 week to 3 months based on clinical conditions and proximity to the hospital. Four general practitioners and four nurses provide services in both outpatient departments. Medications for hypertensive patients are dispensed by hospital pharmacists, and additional hospital services such as laboratory tests and imaging are available. The study was conducted from October 20 to November 30, 2019.

### 2.2. Study Design

This study employed an explanatory sequential mixed-methods design. Initially, an institutional-based cross-sectional design was utilized to assess self-management behavior levels and factors related to self-care among hypertensive patients. Subsequently, a descriptive qualitative interview study design was applied to explore factors related to self-care behaviors among hypertensive patients.

### 2.3. Source and Study Population

The source population included all hypertensive patients on follow-up at Dessie Referral Hospital in Dessie, Ethiopia. The study population consisted of selected patients who met the inclusion criteria and were available during the data collection period.

### 2.4. Eligibility Criteria

#### 2.4.1. Inclusion Criteria

Patients were included in this study, both qualitatively and quantitatively, if they met the following criteria:

• Being hypertensive and attending chronic follow-up at the outpatient department during the study period.
• Having been on hypertension follow-up for at least the last 6 months.
• Age greater than 18 years.
• Proficiency in the Amharic language.

### 2.5. Sample Size Determination

The sample size for the study was calculated using the single population proportion formula, considering a 95% confidence level and a margin of error (0.05). The sample size calculation considered *p* = 0.619, determined by comparing *p* values for medication, exercise, diet, alcohol, smoking, and weight management adherence to identify the largest sample size [1]. A population correction formula was applied, and a 10% nonresponse rate was added.

(1) $$\begin{array}{c} n={\left(\frac{z\propto }{2}\right)}^{2}*p*\frac{\left(1-p\right)}{d^{2}}, \end{array}$$

where

• *n* = minimum sample size
• *z*∝/2 = reliability coefficient for the desired confidence interval (CI) at 95% CI, *z*∝/2 = 1.96
• *p* = 0.619
• *q* = 1 − *p* = 0.381
• *d* = desired precision level (degree of precision) = 0.05 (5%)

(2) $$\begin{array}{c} n=\frac{{\left(1.96\right)}^{2}*\left(0.619\right)*\left(0.381\right)}{{\left(0.05\right)}^{2}}=362. \end{array}$$

The formula yields a sample size of 362 hypertensive patients. Given that the estimated total population of hypertensive patients is less than 10,000, a correction formula was applied.

(3) $$\begin{array}{c} nf=\frac{n}{\left(1+n/N\right)}, \end{array}$$

where

• *nf* = final sample size
• *N *= total targeted population on chronic follow-up in Dessie Referral Hospital (4,612).

Using this equation, the final sample size was determined to be 336, and after adding a 10% nonresponse rate, the final sample size was adjusted to 370.

### 2.6. Sampling Procedure

Dessie Referral Hospital has a total of 4612 hypertensive follow-up patients. The sampling frame consisted of all 370 previously registered patients, who were included in the sampling frame. On average, 25 hypertensive patients visit Dessie Referral Hospital for follow-up each day. The study participants were selected using systematic random sampling. To determine the sampling interval (*k*), the expected number of hypertensive patients attending follow-up in 1 month was divided by the sample size (750/370 ≈ 2). The first respondent was selected using a lottery method from the first two patients who arrived at the hospital on the data collection start date.

For qualitative interviews, participants were selected purposively. Those participants who were assumed to have knowledge regarding barriers to self-care behaviors were selected based on their year of diagnosis and with the assistance of nurses working at the chronic ailments' clinic. Data collection continued until thematic saturation was reached, as saturation is an essential criterion in qualitative research to determine when to discontinue data collection and/or analysis (Saunders, 2018). Fourteen respondents who also participated in the quantitative study were included in the qualitative interviews.

### 2.7. Study Variables

#### 2.7.1. Dependent Variable

• Self-care behaviors for hypertensive control.

#### 2.7.2. Independent Variables

• Sociodemographic factors: age, sex, educational status, occupational status, and address of patients.
• Health profile of the patients: duration of hypertension diagnosis, presence of comorbidity, and family history of hypertension.
• Source of information about self-care: health professionals, friends, books, media, and family.
• Lifestyle factors: medication usage, diet, exercise, alcohol consumption, and tobacco consumption.

### 2.8. Operational Definitions

• Self-care: The ability of individuals, families, and communities to promote health, prevent disease, maintain health, and cope with illness and disability with or without the support of a healthcare provider [9].
• Self-care practice: A fundamental aspect of patient-centered hypertension self-control and care.
• Adherence to self-care behaviors: The extent to which patients adhere to the six types of self-care behaviors, including medication adherence, physical activity, alcohol abstinence, smoking cessation, adherence to a low-salt diet, and weight management [10].
• Medication adherence: The number of days in the last week that an individual takes medication at the recommended dosage and at the same time.
• Diet-related adherence: Participants who reported usually or always consuming a diet rich in grains, fruits, and vegetables; those who rarely or never consumed salt; and those who rarely or never consumed foods rich in spices and saturated fat were considered adherent.
• Exercise-related adherence: Respondents who reported exercising for more than 30 min per day, at least three times per week.
• Smoking-related adherence: Participants who reported either stopping smoking or never having smoked.
• Alcohol consumption–related adherence: Participants who reported never consuming alcohol or who stopped alcohol consumption due to the disease. Respondents who did not consume alcohol at all were considered abstainers.
• Weight management: This involves activities undertaken to manage weight through dietary practices, such as reducing portion sizes and making food substitutions, as well as engaging in exercise for weight loss and agreement with weight management activities over the past 30 days.
• Comorbidities: Respondents with one or more medical conditions in addition to hypertension.

### 2.9. Data Collection Procedure

Both quantitative and qualitative data were collected from hypertensive patients receiving treatment at the outpatient department chronic care clinic of Dessie Referral Hospital. Quantitative data collection took place from October 20 to November 25, 2019, using a structured questionnaire that covered sociodemographic information, self-care behaviors, and factors related to patients' data. The questionnaire was developed after reviewing relevant literature, with necessary modifications. The assessment tool for self-care practices was adapted from Warren-Findlow [10] and a study on lifestyle modification in hypertensive patients by Abel et al. [11]. After obtaining written informed consent, patients were interviewed in Amharic, the local language, in a face-to-face manner. The questionnaire, initially prepared in English, was translated into Amharic by language experts.

Quantitative data were collected by 5 pharmacists, and the data collectors received one-day training on the measurement tools from the principal investigator. Data collectors were supervised by the principal investigator, and data collection continued for 4 weeks.

A pretest of the questionnaire was conducted using two pharmacists for data collection. Five percent of the questionnaires were distributed 1 week before data collection to assess logical sequencing and alignment with the research objectives. This pretest was conducted at Boru Meda Hospital, a facility with similar socioeconomic characteristics to the study population, although it was not the main data collection site.

Qualitative data collection was performed by the principal investigator using semistructured interview guides developed through a review of relevant literature. In-depth interviews were conducted from November 25 to November 30, 2019, and the participants were given the freedom to express additional thoughts and comments. Voice recorders and field notes were used to capture information, and interviews typically lasted about 30 min on average.

### 2.10. Data Analysis Procedure

Data were checked for completeness, cleaned, and then entered into EpiInfo Version 7 before being exported to SPSS Version 24. Incomplete or inconsistent data were excluded from the analysis. Descriptive statistical analyses, such as frequency distribution and means, were used. Bivariate analysis was initially employed to identify factors associated with the dependent variable. Independent variables with associations at a significance level of *p* < 0.25 were included to ensure potentially significant factors were not excluded prematurely. This approach aligns with standard practices. These variables were then entered into a multivariate logistic regression model to assess independent associations, while confounding variables were controlled by including relevant factors in the model. Only variables with statistical significance at *p* < 0.05 were retained in the final model. Odds ratios with 95% CI were used to measure the magnitude of association between independent variables and the dependent variable. Results were presented using charts and tables. Qualitative data from in-depth interviews were transcribed verbatim and analyzed thematically by the primary investigator using thematic analysis. The transcripts were stored and managed to ensure confidentiality, and the finalized transcripts were translated into English by an independent translator. To enhance understanding, the analysis involved multiple readings of the transcripts. Coding was initially conducted in Amharic, translated into English, and then cross-checked for accuracy. Starting with the first interview, transcripts were reviewed line by line during the coding process. Data collection and coding were performed simultaneously with the analysis. Key participant quotes were included to highlight the study's findings. Regular discussions among the study team were held to refine codes and identify emerging themes, ensuring precise results. Ultimately, related categories were combined into broader conceptual themes. Themes and concepts extracted from the data were presented in narratives and triangulated with the quantitative results in the Discussion section.

### 2.11. Data Quality Management

The questionnaire was initially prepared in English, translated into Amharic, and then back-translated into English by another individual to ensure consistency. A pretest of the questionnaire was conducted, and necessary modifications were made based on the specific situation in the study area. Data collectors received regular feedback and corrections, and the accuracy, clarity, and completeness of collected data were monitored carefully.

The transcripts of qualitative data were shared with research participants to confirm the verbatim accurately reflected their experiences. The data were assured by an expert from the Department of Social and Administrative Pharmacy who confirmed the interpretations accurately. Moreover, a conceptual framework was used to guide the study, methodological triangulation (the data collected in the quantitative part and the qualitative part were compared and contrasted), and more than one investigator was involved in this study.

### 2.12. Reflexivity

The principal investigator (A.M.) was considered an insider, which offered both strengths and potential biases in exploring social issues. He was aware of potential insider bias and the inherent conflicting roles and perceptions associated with his status. Efforts were made to mitigate these limitations, including the use of open-ended questions and informal conversations to encourage participants to share their honest opinions and experiences. Nurses were also informed about the study to help identify knowledgeable patients about hypertension.

## 3. Results

### 3.1. Quantitative Findings

#### 3.1.1. Sociodemographic Characteristics of Participants

Out of the 370 hypertensive patients initially planned for inclusion in the study, 364 completed questionnaires, resulting in a response rate of 98.4%. The mean age (±SD) of the respondents was 56.9 (±11.4) years, ranging from 35 to 95 years. Of the respondents, 201 (52.2%) were female, and 139 (38.2%) were illiterate. Two hundred one (55.2%) identified as Muslims, and 334 (91.8%) belonged to the Amhara ethnic group. About 69 (19.0%) respondents were employed, 238 (65.4%) were married, and 257 (70.6%) lived in urban areas (Table 1).

#### 3.1.2. Clinical Characteristics and Source of Information

Of the participants, 226 (62.1%) had been diagnosed with hypertension for less than 5 years. About 206 (56.6%) had no comorbidities, 271 (74.5%) had no family history of hypertension, and 233 (64.0%) did not know their BMI. The majority, 310 (85.2%), received information about hypertension from health professionals (Table 2).

### 3.2. Self-Care Behaviors of Hypertensive Patients

Out of the respondents, 325 (89.3%) did not have a sphygmomanometer at home to check their blood pressure regularly. Among those who checked their blood pressure, 259 (71.2%) reported checking it regularly, with most of them checking it monthly. One hundred sixty-six (45.6%) respondents had their blood pressure checked at the hospital during their appointment. The majority of patients, 228 (62.6%), did not face barriers to checking their blood pressure when needed (Table 3).

#### 3.2.1. Adherence to Self-Care Behaviors

Two hundred eighty-one (77.2%) of the respondents consistently followed their prescribed medication regimen. Additionally, 226 (62.1%) adhered to dietary recommendations, and 235 (64.6%) maintained a regular exercise routine. An overwhelming majority, 349 (95.9%), were nonsmokers or had successfully quit smoking, while 308 (84.6%) abstained from alcohol consumption. Weight management was a priority for 252 (69.2%) of the respondents, and 271 (74.5%) strictly adhered to a low-salt diet (Table 4).

**Figure 1:**
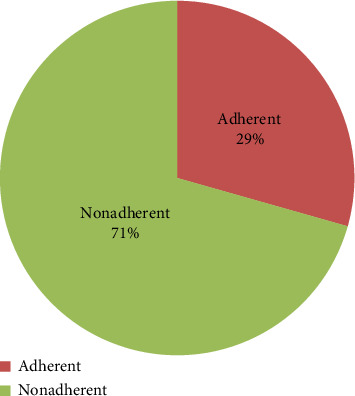
Self-care behaviors of hypertensive patients attending Dessie Referral Hospital, Northeast Ethiopia, 2019 (*N* = 364).

Approximately, one hundred seven (29.4%) respondents adhered to all the prescribed self-care behaviors, encompassing medication compliance, dietary guidelines, regular exercise, smoking cessation, alcohol abstinence, weight management, and low-salt consumption (Figure 1).

#### 3.2.2. Adherence to Diet

Fifty-six (15.3%) participants consistently incorporated fruits into their diet following their hypertension diagnosis. A substantial majority, comprising 297 (81.6%) participants, diligently avoided foods high in saturated fat. Moreover, 236 (64.8%) participants significantly reduced their consumption of spicy foods since their diagnosis. Remarkably, 353 (96.9%) participants demonstrated commendable restraint, with either zero or minimal use of salt in their food preparations (Table 5).

#### 3.2.3. Adherence to Exercise

An overwhelming majority, comprising 333 (91.5%) of the respondents, actively engaged in physical exercise. Among them, 224 (61.5%) diligently exercised a minimum of three times per week, while 232 (63.7%) committed to exercise sessions lasting at least 30 min (Table 6).

### 3.3. Factors Associated With Hypertension Self-Care Behaviors

After controlling for possible confounding effects of other covariates, the duration of hypertension since diagnosis, place of residence, and BMI were found to be significantly associated with adherence to all self-care behaviors.

Respondents residing in urban areas were 70% less likely to adhere than those in rural areas (AOR = 0.3, 95% CI: 0.142, 0.697). Respondents with shorter and longer durations since diagnosis (< 9 years and > 20 years) were significantly associated compared to medium duration (10–19 years) since diagnosis. Respondents with a normal BMI (BMI = 18.5–24) were 47% less likely to adhere than those with abnormal BMI (underweight and overweight) (AOR = 0.526, 95% CI: 0.301, 0.918) (Table 7).

### 3.4. Qualitative Findings

Fourteen in-depth interviews were conducted with participants, comprising 8 males and 6 females. The participants' ages ranged from approximately 35 to 89 years, with a mean age of 52.3 (±13.07) years. Pseudonyms were used for all participants' names (Table 8).

## 4. Qualitative Insights Into Self-Care Behaviors and Lifestyle Practices

The qualitative findings presented in this section offer valuable insights into the self-care behaviors and lifestyle practices of individuals living with hypertension within the study population. Through in-depth interviews, we gained a deeper understanding of how these individuals navigate dietary restrictions, exercise routines, medication adherence, and lifestyle choices to manage their hypertension effectively. Furthermore, this qualitative exploration sheds light on the challenges they encounter in adhering to recommended practices and the innovative strategies they employ to overcome these obstacles. These narratives provide a rich context for understanding the complexities of self-care in the context of hypertension management.

### 4.1. Diet-Related Adherence and Practice

Many participants emphasized the significance of adhering to dietary recommendations given by their healthcare providers, particularly regarding salt intake. Their commitment to following these dietary restrictions was evident in their statements:

Participant Ql001 (Male, 55) expressed, “*Anybody who has hypertension must accept the advice of his doctors and avoid foods with salt*.”

Participant Ql002 (Female, 44) shared her commitment, saying, “*Since I started taking my medication, I stopped consuming salt, tea, and other items that my doctors advised me against*.”

Participant Ql005 (Male, 58) described his transformation, “*Before becoming a hypertensive patient, I used to consume salt, chew khat, and eat whatever I wanted. But now I am forced to stop everything to stay healthy*.”

While participants were dedicated to their dietary restrictions, they encountered challenges, particularly when they were away from home or living in different conditions. These challenges often made it difficult to adhere to their prescribed diets:

Participant Ql001 (Male, 58) expressed his challenge, “*When I leave my house, I can't always find the foods I need, and they don't always cook what I want, so I end up eating whatever is available.”*

Participant Ql012 (Male, 84) faced constraints related to his living conditions and dietary preferences, explaining, “*I live in the church, and I eat whatever the church's followers provide. I can't eat food without salt because I can't tolerate it. My living conditions are not comfortable*.”

To overcome these challenges, participants employed various coping strategies. They often diluted salty foods with water to reduce sodium intake or opted for dishes that did not contain added salt:

Participant Ql007 (Female, 40) mentioned her strategy, “*When I visit other people's houses for events like weddings and ceremonies, if the food is salty, I add water to dilute it*.”

Participant Ql010 (Female, 60) preferred to avoid salty dishes when attending social gatherings, stating, “*When I attend events like weddings and ceremonies away from home, I prefer dishes without added salt. If the food is salty, I simply refuse to eat it. Sometimes, I send my sons to such social gatherings in my place*.”

### 4.2. Physical Exercise Practice

The majority of participants viewed their daily activities as a form of regular physical exercise and incorporated physical activity into their daily routines:

Participant Ql003 (Male, 54) stated, “Now I engage in regular physical exercise, at least three times a week, for more than 30 minutes each time. However, I've been reducing my exercise because I experience leg pain.”

Participant Ql002 (Female, 44), a teacher, shared her routine, “I walk for about one hour each day to reach my workplace. It's like a form of exercise, and it's sufficient for me.”

While participants were dedicated to integrating physical exercise into their lives, they faced obstacles that occasionally hindered their adherence to their exercise routines. These challenges included factors such as work conditions and physical discomfort:

Participant Ql001 (Male, 58) noted, “I used to exercise regularly, but now I find it difficult to do so because of my work conditions.”

Participant Ql005 (Male, 60) cited pain and past surgery as barriers, explaining, “I can't perform regular physical exercises due to pain and a previous surgery. My best physical exercise is slow jogging every day.”

Participants employed various strategies to overcome these challenges and maintain their commitment to physical exercise. Many chose to walk instead of using transportation for short trips, while others considered household chores a form of physical activity:

Participant Ql010 (Female, 60) mentioned her approach, “Most of the time, when I travel from place to place, I avoid using a taxi and walk on foot. I consider it a form of exercise.”

Participant Ql014 (Female, 35) explained her daily routine, “I handle all the household chores by myself without assistance from others, and this serves as my physical exercise.”

### 4.3. Antihypertensive Medication Adherence Practice

The majority of participants expressed their commitment to adhering to their antihypertensive medication regimen, emphasizing the guidance they received from healthcare professionals:

Participant Ql005 (Female, 55) underscored the importance of medication adherence, stating, “Every hypertensive patient must follow their doctors' advice and take their medication without interruption if they want to lead a healthy life.”

Participant Ql013 (Male, 56) shared his dedication to medication adherence, saying, “I adhere to my medication regimen diligently, even during challenging times like a family member's death. I never forget or stop taking my medication.”

Despite their dedication, some participants faced challenges that occasionally led to nonadherence to their medication. These challenges included cultural beliefs, lack of family support, and difficulties related to timing and memory:

Participant Ql001 (Male, 55) discussed the influence of cultural beliefs on his adherence, explaining, “I live in a community where people believe that adapting to the medication is not good. Due to this cultural influence, I stopped taking the medication when my blood pressure decreased and resumed it when it increased. I'm influenced by culture.”

Participant Ql012 (Male, 84), who was blind, encountered issues with medication timing and memory: “I take my medication as prescribed by my doctors and pharmacists in terms of the number of tablets, but I don't follow a strict schedule. I estimate the time because I'm blind, and no one helps me or reminds me.”

Participants adopted various strategies to overcome these challenges and enhance medication adherence. These included visual reminders and family support:

Participant Ql013 (Male, 56) recommended a practical approach for those struggling with medication timing, suggesting, “For hypertensive patients who struggle with remembering medication timing, they can write it down on paper and post it in a visible place, like in front of the living room, where they can see it when entering or leaving.”

Participant Ql014 (Female, 35) relied on the support of her family: “When I forget my medication, my family, including my children and husband, remind me to take it.”

### 4.4. Alcohol Drinking and Smoking Practice

The majority of participants reported that they refrained from smoking and drinking alcohol throughout their lives, especially after starting their antihypertensive medication. For example:

Participant Ql009 (Male, 60) stated, “I don't smoke, and I don't drink alcohol. I have no addictions except for food and water.”

Participant Ql008 (Female, 35) reiterated, “I don't smoke or drink alcohol at all.”

#### 4.4.1. Reasons for Not Stopping Drinking and Smoking

Nevertheless, a few participants faced difficulties in discontinuing alcohol consumption and smoking due to various factors, including peer pressure and concurrent addictions:

Participant Ql001 (Male, 58) acknowledged his current alcohol consumption, albeit in moderation compared to his past habits, and discussed the influence of friends: “I currently drink alcohol, albeit moderately, compared to before starting my medication. I don't smoke, but my problem is that when I meet my friends, they encourage me to drink.”

Participant Ql003 (Male, 54) described his struggle to quit smoking, particularly when using khat: “When I began taking antihypertensive drugs, I quit drinking alcohol, but I couldn't stop smoking cigarettes, especially when I chew khat. The main issue is that I can't stop chewing khat, and that leads to smoking cigarettes.”

Participant Ql012 (Male, 84) mentioned his difficulty in giving up alcohol due to beliefs about its impact on his health: “I don't smoke regularly, but I do drink ‘tella' (local alcohol). I can't stop because I believe my body will dry up, and I won't be able to urinate.”

## 5. Discussion

This study was conducted to evaluate self-care behaviors and their associated factors among hypertensive patients at Dessie Referral Hospital. The assessment of adherence to self-care behaviors and the identification of influencing factors are crucial for the development of effective strategies to manage hypertension, given that adherence to recommended self-care practices is a pivotal aspect of blood pressure control.

Our findings revealed that the overall adherence to self-care behaviors among hypertensive patients, encompassing aspects such as medication compliance, dietary choices, exercise routines, smoking cessation, alcohol consumption cessation, weight management, and low-salt diets, was relatively low at 29.4%. This prevalence is consistent with similar studies conducted at Gondar University (22%) and Addis Ababa public hospitals (23%), all of which reported low adherence rates [12, 13]. In contrast, a study from Saudi Arabia reported a much lower adherence rate of 4.2%. These disparities can be attributed to variations in sample sizes, demographic characteristics, and study designs.

Nonadherence to antihypertensive medications remains a significant challenge in achieving blood pressure control among hypertensive individuals [14]. In this study, we observed that 77.2% of respondents adhered to their antihypertensive medications, which aligns with findings from Israel (95%) but differs from studies in Iran (36.1%) and India (58.1%). Studies conducted in various regions of Ethiopia, including Jimma University Specialized Hospitals (61.9%) and public hospitals in Addis Ababa (66.8%), reported somewhat higher adherence rates, while Aider Comprehensive Specialized Hospital recorded a lower rate of 48.2% [1, 2, 13, 15–17]. These variations may be attributed to differences in sociodemographic factors and levels of awareness about the disease and antihypertensive medications.

In this study, only 4.1% of respondents reported being smokers, while an impressive 84.6% had either quit drinking alcohol or had never consumed it. These findings were corroborated by the qualitative data, where participants discussed their decisions to abstain from smoking and alcohol consumption since beginning hypertension treatment. Similar studies conducted in Brazil (84.7%) and Jimma University Specialized and Teaching Hospital, Ethiopia (84.7%), reported high rates of alcohol cessation. However, studies in Iran (78.9%) and other parts of Ethiopia recorded lower cessation rates, highlighting potential sociocultural influences [18–20].

Regarding dietary habits, 74.7% of respondents incorporated fruits, vegetables, grains, and beans into their diets upon learning about hypertension, with 62.1% adhering to diet-related self-care behaviors. Furthermore, 74.5% adhered to a low-salt diet. These findings are in line with studies conducted in Addis Ababa public hospitals (69.1%), suggesting that participants take their dietary restrictions seriously, even during social events. Nevertheless, other studies reported lower adherence rates in Ayder Comprehensive Specialized Hospital, Mekele Ethiopia (57.5%), and Iran (12.3%) [13, 17, 18].

In terms of exercise, 64.6% of respondents adhered to exercise-related self-care behaviors. While this is higher than findings in Iran (24.5%) and Jimma, Ethiopia (44.9%), it remains lower than the adherence rate observed in public health hospitals in Addis Ababa (90.1%) [1, 2, 13]. However, qualitative data indicated that 91.5% of participants reported engaging in physical exercise, suggesting that many considered their daily activities, such as walking, as a form of exercise.

Our study identified several factors associated with adherence to self-care behaviors. Participants residing in rural areas were more likely to adhere to all recommended self-care behaviors compared to their urban counterparts, which contrasts with findings from Ayder Comprehensive Specialized Hospital, Mekele Ethiopia, where rural residents were less likely to adhere [17]. Additionally, respondents diagnosed with hypertension for shorter (< 9 years) and longer (> 20 years) durations were more likely to adhere to self-care behaviors than those diagnosed for medium durations (10–19 years), echoing similar findings in Addis Ababa [13]. Furthermore, participants with a BMI within the range of 18.5–24 were less likely to adhere to self-care behaviors, consistent with results from Iran [2].

## 6. Practical Implications

From a practical standpoint, these findings have significant implications for hypertensive patients, communities, and healthcare organizations. Self-care behaviors play a pivotal role in preventing and managing hypertension. Adherence to these behaviors can enhance patient satisfaction, improve quality of life, and reduce healthcare resource utilization. This study underscores the importance of continuous education and support for patients and communities to enhance adherence to self-care behaviors and promote better hypertension management. Future interventions should focus on modifying self-care behaviors and providing ongoing support to individuals and communities to improve adherence.

The qualitative findings from this study offer valuable and unique insights into the self-care behaviors of individuals managing hypertension. Participants exhibited a strong commitment to dietary restrictions, exercise, and medication adherence, reflecting their dedication to managing their condition effectively. These findings underscore the importance of patient education and support to enhance adherence to self-care behaviors and improve hypertension management. Additionally, the strategies employed by participants to overcome challenges, such as diluting salty foods and using visual reminders, highlight the resourcefulness of individuals in adapting to their health needs. Healthcare providers and policymakers can use these insights to develop targeted interventions that address the specific challenges and strategies identified by patients to promote better self-care and hypertension control.

This study underscores the necessity for targeted interventions aimed at enhancing adherence to self-care behaviors among hypertensive patients, ultimately leading to improved blood pressure control and a reduction in hypertension-related complications. It is important to recognize the factors influencing adherence, as they can inform the development of effective strategies and interventions to promote better hypertension management.

## 7. Limitations

This study, while valuable, has certain limitations. First, there is the possibility of social desirability bias in responses due to interviewer-administered data collection, which may have influenced participants to provide more socially acceptable answers. Additionally, the study's scope was limited to hypertensive patients who visited healthcare institutions during the study period, potentially excluding those who did not seek medical care. This could introduce selection bias and not capture the self-care behaviors of all hypertensive individuals in the community. Expanding the scope to include a broader population, particularly addressing the urban–rural divide, would improve the generalizability of findings. These approaches would help mitigate the limitations and provide more comprehensive insights into self-care behaviors. Also the study cross-sectional nature and reliance on self-reported data may introduce recall bias. Moreover, the findings may have limited generalizability due to the specific characteristics of the study population and the potential differences between urban and rural settings. For future research, longitudinal designs are recommended to establish causality and address recall bias. Employing objective measures, such as biometric data, could enhance accuracy.

## 8. Conclusion

This study reveals a significant concern regarding the low adherence to recommended self-care behaviors among hypertensive patients. Key determinants of adherence include the duration since hypertension diagnosis, place of residence, and BMI, while various challenges such as living conditions, work environments, cultural influences, and peer pressure impact adherence. To address this issue effectively, healthcare providers should prioritize comprehensive patient and community education during follow-up sessions, emphasizing behavior modification and continuous support. Patients, in turn, are encouraged to actively seek medical advice, stay informed through reliable sources, and minimize factors hindering adherence. Moreover, further research should delve into organizational, healthcare provider, and patient–provider communication factors related to hypertension self-care behavior adherence. By implementing these recommendations, the management of hypertension can be significantly improved, leading to better health outcomes for patients.

## Figures and Tables

**Table 1:** Sociodemographic characteristics of hypertensive patients who were attending at Dessie Referral Hospital, Northeast Ethiopia, 2019 (*N* = 364).

Variables		Frequency	Percent
Sex	Female	201	52.2
	Male	163	44.8

Age	35–55	178	48.9
	56–75	173	47.5
	+76	13	3.6

Education	Not read and write	139	38.2
	Read and write	70	19.2
	Primary (1–8)	54	14.8
	Secondary (9–10)	22	6.1
	Tertiary (11-12)	16	4.4
	Diploma and above	63	17.3

Religion	Orthodox	157	43.1
	Muslim	201	55.2
	Protestant	5	1.4
	Others	1	0.30

Ethnicity	Amhara	334	91.8
	Oromo	16	4.4
	Tigray	13	3.6
	Others	1	0.3

Marital status	Married	238	65.4
	Single	16	4.4
	Divorced	33	9.1
	Widowed	77	21.2

Occupation	Employed	69	19.0
	Unemployed	59	16.2
	Merchant	35	9.6
	Farmer	69	19.0
	Retire	55	15.1
	House wife	77	21.2

Residence	Urban	257	70.6
	Rural	107	29.4

**Table 2:** Clinical characteristics and source of information of hypertensive patients in Dessie Referral Hospital, Northeast Ethiopia, 2019 (*N* = 364).

Variables		Frequency	Percent
Since diagnosis (years)	< 5	226	62.1
	5–9	73	20.1
	10–14	35	9.6
	15–19	22	6.0
	> 20	8	2.2

Presence of other disease	Yes	158	43.4
	No	206	56.6

Family history of HTN	Yes	93	25.5
	No	271	74.5

Body mass index (BMI)	< 18.5	11	3.0
	18.5–24	107	29.4
	24–30	9	2.5
	> 30	4	1.1
	I do no	233	64.0

Source of information about HTN	Health professionals	310	85.2
	Social media	35	9.6
	Books	10	2.7
	Family and friends	7	1.9
	No information	2	0.50

**Table 3:** Self-care behaviors of hypertensive patients attending Dessie Referral Hospital, Northeast Ethiopia, 2019 (*N* = 364).

Variables		Frequency	Percent
Do you have a home sphygmomanometer?	Yes	39	10.7
	No	325	89.3

Do you regularly check your BP?	Yes	259	71.2
	No	105	28.8

If yes, how often do you check your BP?	Daily	23	6.3
	Weekly	33	9.10
	Monthly	166	45.6
	Bimonthly	34	9.3
	Every 3 months	2	0.5
	More than that	1	0.3

If yes, from where do you get your BP checked?	At home	26	7.1
	At hospital	166	45.6
	Nearest health care facility	36	9.9
	Near pharmacy	31	8.5

What are the barriers toward self-testing for BP?	Expensive	57	15.7
	Lack of awareness	65	17.9
	Pain	11	3.0
	Don't feel the need	3	0.80
	None	228	62.6

**Table 4:** Adherence to self-care behaviors of hypertensive patients attending Dessie Referral Hospital, Northeast Ethiopia, 2019 (*N* = 364).

Variables		Frequency	Percent
Medication usage	Adherent	281	77.2
	Nonadherent	83	22.8

Diet-related adherence	Adherent	226	62.1
	Nonadherent	138	37.9

Exercise-related adherence	Adherent	235	64.6
	Nonadherent	129	35.4

Smoking	Ceased	31	8.5
	Not cease	15	4.1
	Totally not smoking	318	87.4

Alcohol consumption	Moderated	40	11.0
	Not moderated	16	4.4
	Not drinking	230	63.2
	Ceased	78	21.4

Weight management	Adherent	252	69.2
	Nonadherent	112	30.8

Low-salt diet	Adherent	271	74.5
	Nonadherent	93	25.5

**Table 5:** Answers of participants on diet-related practices in Dessie Referral Hospital, Northeast Ethiopia, 2019 (*N* = 364).

Variables	Never	Rarely	Usually	Always
How often do you include fruits, vegetables, grains, and beans in your diet after diagnosis?	36 (9.9%)	272 (74.7%)	50 (13.7%)	6 (1.6%)
How often do you consume foods that contain high saturated fat?	297 (81.6%)	59 (16.2%)	8 (2.2%)	0
How often do you consume spicy foods since being diagnosed?	56 (15.4%)	236 (64.8%)	66 (18.1%)	6 (1.6%)
How often do you consume salt in your food?	125 (34.3%)	228 (62.6%)	11 (3.0%)	0

**Table 6:** Response of study participants on performance of exercise-related activities in Dessie Referral Hospital, Northeast Ethiopia, 2019 (*N* = 364).

Variables		Frequency	Percent
Do you perform physical exercise at all?	Yes	333	91.5
	No	31	8.5

How often do you exercise?		224	61.5
	≥ 3 times per week	109	29.9

For how long do you exercise per session?		232	63.7
	≥ 30 min per day	101	27.7

**Table 7:** Factors associated with hypertension self-care behaviors of participants in Dessie Referral Hospital, Northeast Ethiopia, 2019 (*N* = 364).

Variables		All self-care behavior adherent		COR, 95% CI	*p* value	AOR, 95% CI	*p* value
		Adherent	Nonadherent				
Occupations	Employed	25	44	0.660 (0.321–1.331)	0.246	0.734 (0.320–1.684)	0.466
	Unemployed	12	47	0.656 (0.312–1.381)	0.267	0.735 (0.321–1.681)	0.466
	Merchant	12	23	1.469 (0.654–3.296)	0.351	1.477 (0.608–3.584)	0.388
	Farmer	17	52	1.147 (0.546–2.41)	0.451	0.453 (0.179–1.145)	0.095
	Retire	20	35		0.27		0.280
	House wife	21	56	0.719 (0.304–1.698)	0.451	0.745 (0.285–1.941)	0.548

Place of residence	Urban	89	168	0.382 (0.216–0.674)	0.001	0.30 (0.142–0.697)^∗∗∗^	0.002
	Rural	18	89				

Since diagnosis (years)	< 5	56	170	12 (1.98–72.736)	0.007	13.872 (2.159–89.211)^∗∗∗^	0.006
	5–9	30	43	9.107 (1.787–46.414)	0.008	12.48 (2.27–63.594)^∗∗∗^	0.004
	10–14	7	28	5.25 (0.85–32.43)	0.074	6.033 (0.867–41.984)	0.069
	15–19	8	14	4.3 (0.812–22.77)	0.086	4.536 (0.816–25.211)	0.084
	> 20	6	2		0.005	^∗∗∗^	0.001

Presence of other disease	Yes	41	117				
	No	66	140	1.345 (0.849–2.132)	0.207	1.214 (0.73–2.02)	0.45

Body mass index	< 18.5	4	7	2.33 (0.107–50.982)	0.590	0.414 (0.101–1.702)	0.222
	18.5–24	40	67	3.375 (0.189–60.238)	0.408	0.526 (0.301–0.918)^∗∗^	0.024
	24–30	4	5		0.790		0.129
	> 30	2	2	2.37 (0.147–38.29)	0.543	0.579 (0.153–2.196)	0.422
	I do no	57	176	1.333 (0.057–31.121)	0.858	0.243 (0.032–1.876)	0.175

*Note:* Statistically significant (*p* < 0.05), AOR, adjusted odds ratio for multivariate logistic regression, only those independent variables were used in the model that were significant in bivariate analysis, and the cutoff point was *p* < 0.25.

Abbreviations: CI, confidence interval; COR, crude odds ratio.

^∗∗^ *p* value < 0.05.

^∗∗∗^ *p* value < 0.01.

**Table 8:** Personal characteristics of qualitative interview participants in chronic follow-up units of Dessie Referral Hospital, Northeast Ethiopia, 2019 (*n* = 14).

Codes	Age	Gender	Marital status	Religion	Occupation	Duration of HTN
Ql001	58	Male	Married	Christian	Merchant	5 years
Ql002	44	Female	Married	Muslim	Teacher	3 years
Ql003	54	Male	Married	Christian	Guard	8 years
Ql004	55	Male	Married	Christian	Farmer	2 years
Ql005	55	Female	Married	Christian	Cleaner	5 years
Ql006	39	Female	Married	Muslim	Housewife	3 years
Ql007	40	Female	Widowed	Muslim	Merchant	6 months
Ql008	35	Female	Married	Muslim	Housewife	2 years
Ql009	60	Male	Married	Muslim	Teacher	19 years
Ql010	60	Female	Widowed	Christian	Housewife	5 years
Ql011	56	Male	Married	Muslim	Farmer	1 year
Ql012	84	Male	Single	Christian	Priest	3 years
Ql013	59	Male	Married	Christian	Teacher	7 years
Ql014	35	Male	Single	Christian	Unemployed	1 year

## Data Availability

The datasets generated during and/or analyzed during the current study are available from the corresponding author on reasonable request.
